# Influence of NKG2D Genetic Variants on Response to Anti-TNF Agents in Patients with Rheumatoid Arthritis

**DOI:** 10.3390/genes9020064

**Published:** 2018-01-25

**Authors:** Milena Iwaszko, Jerzy Świerkot, Katarzyna Kolossa, Sławomir Jeka, Piotr Wiland, Katarzyna Bogunia-Kubik

**Affiliations:** 1 Laboratory of Clinical Immunogenetics and Pharmacogenetics, Hirszfeld Institute of Immunology and Experimental Therapy, Polish Academy of Sciences, Weigla 12, 53-114 Wrocław, Poland; bogunia@iitd.pan.wroc.pl; 2 Department of Rheumatology and Internal Medicine, Wrocław Medical University, Borowska 213, 50-556 Wrocław, Poland; jurekswierkot0@poczta.onet.pl (J.Ś.); pwiland1@gmail.com (P.W.); 3 Clinical Department of Rheumatology and Connective Tissue Diseases, Hospital University Number 2 Jana Biziela, Ujejskiego 75, 85-168 Bydgoszcz, Poland; katarzynakolossa@wp.pl (K.K.); s.jeka@wp.pl (S.J.); 4 Department of Internal, Occupational Diseases, Hypertension and Clinical Oncology, Wrocław Medical University, Borowska 213, 50-556 Wrocław, Poland

**Keywords:** *NKG2D* polymorphism, anti-TNF therapy, TNF inhibitors

## Abstract

A natural killer group 2 member D (NKG2D) acts as a powerful activating and co-stimulatory receptor on immune effector cells including NK and T cells. Disruptions within the NKG2D signalling pathway may trigger an exacerbated immune response and promote autoimmune reactions. The objective of the study was to evaluate a plausible role of polymorphisms within the *NKG2D* gene as a predictor of how effective anti-tumor necrosis factor (TNF) therapy is in rheumatoid arthritis (RA) patients. A total of 280 RA patients receiving anti-TNF therapy were genotyped for *NKG2D* rs2255336 (A > G), rs1049174 (C > G), and rs1154831 (C > A). Clinical response was evaluated according to the European League against Rheumatism (EULAR) criteria at the 12th and 24th week. Both the *NKG2D* rs225336 and rs1049174 polymorphisms were significantly associated with efficacy of TNF inhibitors. Inefficient therapy was more frequently observed in patients with rs2255336 GG or rs1049174 CC genotype as compared to other genotypes (*p*-value = 0.003 and *p*-value = 0.004, respectively). The presence of the rs2255336 G or the rs1049174 C allele correlated with a worse EULAR response (*p*-value = 0.002, *p*-value = 0.031, respectively). Moreover, patients carrying the rs2255336 or rs1049174 heterozygous genotype achieved better EULAR responses than patients with homozygous genotypes (*p*-value = 0.010 and *p*-value = 0.002, respectively). Data from the present study provides evidence that *NKG2D* polymorphisms may affect response to anti-TNF inhibitors in RA patients.

## 1. Introduction

Rheumatoid arthritis (RA) represents one of most common autoimmune disorders, affecting approximately 1% of the worldwide population. The exact cause of RA is not fully understood. However, a combination of genetic and environmental factors underlies the initiation and continuation of RA pathology. An involvement of a genetic component in RA development is estimated to be around 50% [1,2].

Significant progress in RA management has been achieved after introducing anti- tumor necrosis factor (TNF) biologic agents to clinical practice [3]. However, a considerable discrepancy in patients’ responses to the treatment constitutes an important limitation in this approach. Therapy with TNF inhibitors is ineffective in up to 30% of patients [4,5]. The variety of therapeutic effects related to anti-TNF agents may reflect individual genetic backgrounds of patients. Genetic factors may be involved in determining the response to anti-TNF treatment. A selection of patients to anti-TNF therapy may be optimized by identifying those with a decreased likelihood to benefit from the therapy. Pharmacogenomic biomarkers may constitute a powerful tool for predicting therapy outcomes and contributing to considerable improvement of anti-TNF efficacy as well as minimizing adverse effects and costs of treatment [6,7].

An essential role in RA pathophysiology has been attributed to T lymphocytes, as well as natural killers (NK) cells [8,9,10,11]. Inadequate signalling transduced by a repertoire of activatory and inhibitory receptors presented on NK and T cells’ surfaces may lead to deregulated functions of these cells and contribute to the promotion and continuation of RA pathology. Among a broad array of activating receptors, the important role in balancing T and NK effector responses is exerted by the natural killer group 2 member D (NKG2D) receptor belonging to the C-type lectin like family of transmembrane proteins [12,13]. The NKG2D receptor is encoded by the killer cell lectin-like receptor subfamily K member 1 (KLRK1) gene located on chromosome 12 within the natural killer group 2 (NKG2) complex [14]. This receptor is expressed as homodimer on a cell surface of all NK cells, as well as on CD8+ T cells and γδ T cells [15,16,17]. Since NKG2D contains no signalling motifs within its intracellular domain, it associates with DNA X-activating protein of 10 kDa (DAP10) indispensable for signal transduction [18,19]. The NKG2D molecule functions as a powerful activating and co-stimulatory receptor of NK and T lymphocytes involved in recognizing and eliminating dysfunctional cells by interacting with specific ligands [20]. This receptor binds to several diverse ligands structurally homologous to major histocompatibility complex (MHC) class I molecules belonging to two families of cell surface glycoproteins called the MHC class I-chain related proteins (MICA and MICB) and the UL-16 binding proteins (ULBP) [15,21]. These molecules display limited expression on healthy cells and are upregulated when exposed to pathogen infection, tumorigenesis, or cellular stress [22,23]. The NKG2D–ligand system acts as a key regulator of microbial and tumor immunosurveillance [24,25]. Dysregulation of this signalling pathway may lead to inadequate NK and T cell activation and contribute to initiating or maintaining an inflammatory cascade, resulting in self-reactivity [20,26,27]. The NKG2D-mediated signalling pathway has been implicated in RA pathogenesis [28]. Furthermore, a beneficial effect of the NKG2D blockade was observed in a study based on a mouse model of RA (collagen induced arthritis (CIA)), as well as in other autoimmune disorders [29,30,31].

In accordance to our knowledge, there are no pharmacogenetic studies published to date regarding the plausible role of *NKG2D* genetic variants in balancing anti-TNF treatment outcomes. The objective of the present study was to evaluate a potential of *NKG2D* polymorphisms to act as a genetic predictor of clinical response when patients with RA are treated with TNF inhibitors. 

## 2. Materials and Methods

### 2.1. Patients

The study involved 280 patients diagnosed according to the American College of Rheumatology 1987 revised criteria for RA and qualified for anti-TNF therapy. All patients were characterized with a presence of active disease (defined as a Disease Activity Score in 28 joints [DAS28] ≥ 5.1) prior to starting anti-TNF therapy and were resistant to treatment with at least two disease-modifying anti-rheumatic drugs (DMARDS). The inclusion criteria also included: patients over 18 years of age, Caucasian origin, and a complete medical history and physical examination of patients. The following exclusion criteria for selecting patients for anti-TNF therapy were applied in the present study: clinically significant impairment of hepatic and renal function; coexistence of other systemic diseases of connective tissue besides RA; infection with hepatotropic viruses; infections resistant to therapy; ongoing history of cancer or uncontrolled diabetes; alcohol abuse; pregnancy or breastfeeding; insufficient clinical records; and unwillingness or inability to cooperate. Clinical data obtained from the patients included: level of C-reactive protein (CRP), erythrocyte sedimentation rate (ESR), presence of rheumatoid factor (RF) and anti-cyclic citrullinated peptide antibodies (anti-CCP), disease activity score (DAS28), painful and swollen joint counts, global health assessments by a patient and a physician, and a visual analogue scale (VAS, range, 0 to 100 mm) of pain. The study was approved by the Wrocław Medical University Ethics Committee (identification code KB-599/2012) and written informed consent was obtained from all participants.

### 2.2. Treatment Protocol

The patients were treated with recommended doses of anti-TNF agents, which include 3 mg/kg of body weight of infliximab given as intravenous infusions during weeks 0, 2, and 6, and every eight weeks thereafter. Subcutaneous injections of adalimumab at 40 mg every other week, subcutaneous injections of etanercept at 50 mg every week, and subcutaneous injections of certolizumab pegol 400 mg at weeks 0, 2, and 4 and 200 mg every two weeks thereafter. In most cases DMARDs, glucocorticoids, and/or non-steroidal anti-inflammatory drugs were maintained at stable levels as a concurrent therapy. In total 92% of patients received methotrexate (mean dose: 22 mg per week) and 91% of patients were treated with glucocorticoids (mean dose of prednisone: 6.6 mg per day).

### 2.3. Evaluation of Anti-TNF Treatment Outcome

Disease activity of RA patients was evaluated using the DAS28 score based on four components, which included the number of swollen and tender joints, levels of CRP and ESR, and patients’ global assessment of general health expressed on the visual analogue scale (VAS, mm). The level of disease activity was interpreted as low (DAS28 ≤ 3.2), moderate (3.2 < DAS28 ≤ 5.1) or high (DAS28 > 5.1).

The clinical outcome of anti-TNF therapy was assessed according to the European League Against Rheumatism (EULAR) response criteria based on combining an individual amount of change in a DAS28 score and the DAS28 score attained at the given endpoint during the 12th and 24th week after starting treatment [32]. The anti-TNF treatment response calculations were performed at the 12th and 24th week after starting therapy. The patients were categorized as good, moderate, or non-responders. A good response was defined as improvement in a DAS28 score (ΔDAS28) > 1.2 from the baseline in combination with a DAS28 ≤ 3.2 at the endpoint. The moderate response was defined as ΔDAS28 > 1.2 and DAS28 at endpoint > 3.2 or 0.6 < ΔDAS28 ≤ 1.2 and DAS28 at endpoint ≤ 5.1. A lack of response was defined as ΔDAS28 ≤ 0.6 or 0.6 < ΔDAS28 ≤ 1.2 and DAS28 at endpoint > 5.1.

### 2.4. Single Nucleotide Polymorphisms Selection and Genotyping

For the present study, the selection of genetic variants within the *NKG2D* gene was based on available literature analysis as well as search results from HapMap Project and NCBI Database of Short Genetic Variations (dbSNP). Information regarding predicted functional consequences of SNPs was obtained using the SNPinfo Web Server [33]. All studied SNPs were characterized with minor allele frequencies above 10% (1000 Genomes Project) [34].

The following three SNPs in the *NKG2D* gene were selected for analysis: 3′ untranslated region (UTR) genetic variant rs1049174 (C > G) located in the presumed miRNA binding site; rs2255336 A > G (Thr72Ala) nonsynonymous polymorphism positioned in exon 4 near the intron–exon junction region with potential to affect splicing; rs1154831 (C > A) intronic variant encompassing the putative transcription factor binding site. Assessments of linkage disequilibrium (LD) between the SNPs were performed by taking into account data sampled for this study as well as population data available from the 1000 Genomes Project. The LD in sampled data was calculated using R ‘genetics’ package (version 1.3.8.1, https://cran.r-project.org/web/packages/genetics) and the population’s LD was acquired from the LD Calculator (https://caprica.genetics.kcl.ac.uk/~ilori/ld_calculator.php). In either case, the resulting R^2^ values were below 0.5.

Genomic DNA was isolated from peripheral blood taken on EDTA using a Maxwell 16 Blood DNA Purification Kit (Promega Corp., Madison, WI, USA) following the recommendation of the manufacturer. Genotyping of *NKG2D* rs1154831 and rs1049174 genetic variants was performed by real-time polymerase chain reaction (PCR) amplifications and a melting-curve analysis using a LightSNiP typing assay (TIB-MolBiol, Berlin, Germany). The *NKG2D* rs2255336 polymorphism was studied using a PCR-based predesigned TaqMan SNP genotyping assay (Life Technologies, Paisley, UK). The real-time PCRs were carried out on a LightCycler 480 Real-Time PCR system (Roche Applied Science, Penzberg, Germany) for *NKG2D* rs1154831 and rs1049174 as well as a 7300 Real Time PCR System (Applied Biosystems, Foster City, CA, USA) for *NKG2D* rs2255336 in accordance to the conditions recommended by the manufacturers.

### 2.5. Statistical Analysis

The main clinical and demographic features of patients were expressed as continuous variables using mean and standard deviation or as categorical data using frequencies and proportions. Baseline clinical parameters of RA patients were analysed for a potential association with the investigated SNPs using Fisher’s exact test for parametric variables or the Mann–Whitney test for quantitative data. Response to therapy with TNF inhibitors was evaluated in accordance to EULAR criteria at two different time points: the 12th and 24th week after starting the treatment. The patients were categorized into good, moderate, or non-responders. Association of the examined SNPs with a clinical outcome of anti-TNF treatment were assessed by comparing the EULAR scores between the allele and the genotype groups using Fisher’s exact test. A *p*-value < 0.05 was considered statistically significant. Reconstruction of haplotypes comprising three SNPs within the *NKG2D* gene was performed using PHASE software (version 2.1.1. Linux; http://stephenslab.uchicago.edu/software.html#phase). The software employs a method based on the Bayesian coalescent theory and incorporates a model that allows for recombination and decay of linkage disequilibrium with physical distance of alleles [35,36]. The frequencies of haplotypes among RA patients in relation to the anti-TNF response were assessed by using Fisher’s exact test. All statistical calculations were performed in an R software environment (version 3.2.3) [37].

## 3. Results

### 3.1. Patients’ Baseline Characteristics and Response to Anti-TNF Treatment

The main demographic and baseline clinical features of the patients are presented in Table 1.

Among the 280 patients included in the study, 78.6% were female. The mean ± Standard Deviation (SD) age of the RA patients was 51.6 ± 12.3 years, the disease duration was 12.5 ± 8.1 years, and age at disease onset was 39.3 ± 12.0 years. There were 65.9% of RF-positive patients and, in 95.4% of the RF-positive patients, the presence of antiCCP–antibodies was detected.

In accordance to EULAR criteria, good response to anti-TNF therapy after 12 weeks was achieved by 10.4% of patients and moderate response was achieved by 81.4% of patients. However, 8.2% of patients did not respond to treatment. After 24 weeks of therapy, 48.1% of patients were characterized with good response and 46.7% with moderate response while a lack of response was observed in 5.2% of patients.

### 3.2. Linkage Disequilibrium between the NKG2D Polymorphisms

All three studied SNPs were found to be in linkage disequilibrium both a sample- and population-wise. The scaled LD estimate (D’) from patients’ data range between 51% (rs1154831/rs2255336) and 96% (rs1049174/rs2255336). Population-wise, the respective values span 93% to 96%, respectively. However, these SNPs have not been considered good predictors of each other since squared correlation coefficients (R^2^) range from 0.014 (rs1154831/rs2255336) to 0.272 (rs1049174/rs2255336) sample-wise and from 0.053 (rs1154831/rs2255336) to 0.466 (rs1049174/rs2255336) in population data.

### 3.3. Effect of NKG2D Genetic Variants on Anti-TNF Treatment Response

The efficacy of therapy with TNF inhibitors in relation to the *NKG2D* genetic variants is presented in Table 2. A statistically significant association was observed between the *NKG2D* rs1049174 and response to treatment after 12 weeks of therapy with anti-TNF agents. The analysis of the alleles distribution revealed that the C allele was overrepresented among patients characterized with no response to treatment (odds ratio (OR) = 2.39, confidence interval (CI) = 1.07–6.05, *p* = 0.031). Taking into account the *NKG2D* rs1049174 genotypes’ distributions, the presence of the CC genotype in patients correlated with anti-TNF treatment failure when compared to the other genotypes (OR = 4.02, CI = 1.45–12.87, *p* = 0.004). Moreover, the heterozygous CG genotype was associated with better responses to TNF blockade therapy. The good/moderate response was more frequently observed in patients carrying the heterozygous genotype than patients with the homozygous genotypes (OR = 4.76, CI = 1.54–20.00, *p* = 0.002).

There were no significant differences in allele and genotype distributions between the *NKG2D* rs1154831 polymorphism and response to treatment with TNF inhibitors. 

With respect to the *NKG2D* rs2255336, a significant association with the clinical outcome at the 12th week of therapy with TNF inhibitors was also detected. A lack of response was more frequently observed among patients carrying the GG genotype than in patients with the AA or AG genotype (OR = 6.88, CI = 1.62–61.80, *p*-value = 0.003). This effect was accompanied by a significant increase in frequency of the G allele among patients not responding to anti-TNF treatment (OR = 6.26, CI = 1.59–54.05, *p*-value = 0.002). On the other hand, patients with the heterozygous AG genotype achieved significantly better EULAR responses when compared to patients bearing the homozygous GG or AA genotypes (OR = 5.56, CI = 1.33−50.00, *p*-value = 0.010). Regarding a potential association of AA homozygosity with an anti-TNF clinical outcome, the statistical analysis could not be performed as there were no AA carriers among patients not responding to treatment.

### 3.4. Distribution of the NKG2D Alleles and Genotypes with Regard to Selected Clinical Parameters

The relationships of the *NKG2D* rs2255336, rs1049174, and rs1154831 polymorphisms with baseline clinical parameters of the disease, comprising RF status, presence of CCP-antibodies, and DAS28 and CRP values were also assessed (Table 3). There were no significant differences of the *NKG2D* rs2255336, *NKG2D* rs1049174, or *NKG2D* rs1154831 genotype and allele distributions in relation to the presence of the rheumatoid factor. Also, the genotype and allele frequencies of the examined genetic variants did not differ between anti-CCP positive and anti-CCP negative patients. Furthermore, with respect to DAS28 and CRP values prior to anti-TNF treatment, no significant associations with the investigated SNPs were found.

### 3.5. Distribution of the NKG2D Alleles and Genotypes with Regard to EULAR Responses to Adalimumab as well as Etanercept Treatment at 12th Week

Stratification of patients’ cohort according to administered anti-TNF agents was also performed (Table 4). Recipients of infliximab and certolizumab pegol were not analyzed separately due to a small number of patients. 

Analysis involving patients treated with adalimumab did not reveal any significant associations between *NKG2D* polymorphisms and clinical response. However, a statistically significant relationship of the *NKG2D* rs2255336 polymorphism was seen in relation to the etanercept response. Among the etanercept-treated patients, presence of the GG genotype was associated with a lack of response to anti-TNF treatment after 12 weeks (OR = ∞, CI = 1.19 –∞, *p*-value = 0.021). Furthermore, the frequency of the A allele decreased among recipients of etanercept that did not respond to the therapy (OR = 0.00, CI = 0.00–0.82, *p*-value = 0.027). In addition, a relationship between the AG genotype and a good etanercept response was observed on a border of significance (OR = 0.00, CI = 0.00–1.12, *p*-value = 0.051). With regard to the *NKG2D* rs1049174 genetic variant, no correlation with the clinical outcome within the etanercept-treated subgroup was detected.

### 3.6. Analysis of NKG2D Haplotypes

The haplotype structure was as follows: rs1049174 (C > G)-rs2255336 (A > G)-rs1154831 (C > A). In total, seven haplotypes were identified, but only the four most frequent (CGC, GAC, CGA, GGC) that accounted for 96.9% of the total observations (48.0, 17.0, 16.3, and 15.6%, respectively) were incorporated for further analysis (Table 5). A significant association was observed between the GAC haplotype and the clinical outcome of anti-TNF treatment in RA patients at the 12th week. There was a significantly decreased frequency of the GAC haplotype among patients exhibiting lack of response at the 12th week in comparison to other haplotypes (OR = 0.08, CI = 0.00–0.52, *p*-value = 0.001). Furthermore, the significant relationship of the GGC haplotype with response to anti-TNF agents has been detected. The presence of the GGC haplotype correlated with a better outcome of TNF blockade therapy among RA patients (OR = 3.54, CI = 1.28–11.33, *p*-value = 0.008).

## 4. Discussion

The NKG2D molecule, acting as a powerful activating and co-stimulatory receptor on various immune effector cells, contributes to maintaining balance in the immune network of stimulatory and inhibitory signals. Involving the NKG2D receptor in inducing NK and T cells implies an important role of this receptor in both innate and adaptive immunity. The NKG2D-ligand interaction represents a crucial defence mechanism leading the elimination of dysfunctional cells [12,17]. However, inadequate stimulation of this signalling cascade may disrupt self-tolerance and promote an autoimmune response [12,20].

The NKG2D receptor displays the capacity to directly trigger NK cells [15,16,38] and acts as a costimulatory molecule for T cell effector responses [39,40]. The activating signal, transduced via the NKG2D-ligand pathway, may induce the cytotoxic activity of immune effector cells, as well as secretion of proinflammatory cytokines, including TNF-α [15,16,38,41]. On the other hand, proinflammatory cytokines, mainly interleukin-15 (IL-15) and TNF-α, act as modulators of NKG2D expression by inducing increased levels of this protein [28,42]. When serum and inflamed synovium of RA patients are characterized with enhanced levels of IL-15 and TNF-α, NK, and T lymphocytes bearing NKG2D molecules are permanently exposed to high amounts of these proinflammatory cytokines [20,28]. Upon chronic stimulation using IL-15, the NKG2D-mediated pathway may directly trigger effector T cell responses in a manner that is independent of the T cell receptor (TCR) [43].

Enhanced levels of NKG2D are accompanied by abnormal expression of major immunogene complex (MIC) ligands on RA synovial tissue [28]. Additionally, synoviocyte-derived soluble MICAs are present in abundance in the sera of RA patients [20,28]. The expression of NKG2D may be regulated via mechanisms involving ligand-induced downmodulation of the NKG2D. Studies on humans and mice indicate that chronic exposure to soluble and cell surface bound MICA/B ligands result in down-modulation of NKG2D levels, and, subsequently, impairs the NKG2D-dependent signalling pathway in order to prevent chronic NK or T cell stimulation [44,45]. In RA patients, MIC levels are also elevated in serum. Nonetheless, this self-regulatory mechanism is not observed and overexposure to MIC ligands is not associated with decreased levels of NKG2D [28]. The presence of excessive levels of TNF-α and IL-15 pro-inflammatory cytokines in inflamed synovium may act as a counterbalance to ligand-induced downmodulation by constantly upregulating *NKG2D* expression, which may override this mechanism [26,28].

An unusual subset of CD4^+^ T effector cells, described as CD4^+^CD28^−^ T cells, is present in high frequency in peripheral blood and synovium of patients with RA [46]. This population encompasses highly-differentiated effector memory CD4^+^ T cells, and is usually absent in healthy individuals. Pathogenic features, such as cytotoxic activity, secretion of large amounts of cytokines (mainly interferon γ (IFN-γ) and TNF-α), and resistance to apoptosis [46,47,48] have been attributed to these cells. The CD4^+^CD28^−^ T autoreactive cells display tissue-infiltrating ability and are characterized by a lack of the CD28 costimulatory receptors on their surfaces [49]. A significant proportion of these cells acquire the expression of the NKG2D receptor that acts as a co-stimulator of TCR-dependent auto reactivity [28]. A high frequency of CD4^+^CD28^–^ T cells has been also associated with the presence of extra-articular manifestations and advanced joint destruction [50,51]. Clonal expansion of CD4^+^CD28^−^ T cells is also detected in a peripheral circulation in other immune mediated disorders, such as multiple sclerosis [52], inflammatory bowel disease [53], and systemic lupus erythematosus (SLE) [54]. In view of the fact that the CD4^+^CD28^−^ T cell population exhibits high proinflammatory activity and pathogenic potential to induce tissue destruction, these cells may participate in the pathogenesis of autoimmunity [55].

Inadequate expression or conformational changes of the NKG2D receptor, caused by polymorphisms located within its gene, may result in the NKG2D-dependent pathway’s disruption and promote autoimmune pathology. The *NKG2D* rs2255336 SNP is located in the protein’s transmembrane region, close to the binding site of the DAP10 [56], which suggests that this polymorphism may influence NKG2D’s binding affinity to DAP10. Since the DAP10 adaptor protein is indispensable for NKG2D activation, impaired interaction between these proteins may lead to diminished signal transduction and deregulation of NKG2D functions. Proteins encoded by the *NKG2D* rs2255336 genetic variants may display different binding affinities to DAP10, which may result in different potentials of NKG2D activation upon ligand binding. In the present study, the *NKG2D* rs2255336 polymorphism is correlated with efficacy of anti-TNF treatment among RA patients. Inefficiency of anti-TNF therapy was more frequently observed among patients with the GG genotype than in patients bearing the A allele. Additionally, patients carrying the heterozygous genotype achieved significantly better responses to anti-TNF agents when compared to patients with homozygous genotypes. It might be hypothesized that this substitution, resulting in NKG2D proteins with various functional properties, may affect treatment outcomes by balancing NK or T cell functions. 

The *NKG2D* rs2255336 SNP has been shown to modulate natural cytotoxic activity [57]. The *NKG2D* rs2255336 GG genotype was associated with diminished NK cytotoxic activity. Moreover, the GG genotype correlated with an increased overall cancer risk, which provides evidence that *NKG2D* rs2255336 may also be associated with susceptibility to cancer development [57]. The results from a study conducted by Hayashi et al. indicated that *NKG2D* rs2255336 may contribute to determining NK cell function by influencing their cytotoxic activities. In line with these findings, a study regarding cervical cancer observed the protective role of the AA genotype in disease development [58]. To date, there have only been a few studies investigating a possible role of this polymorphism in the development of autoimmune disorders. Previously-conducted studies concerning SLE pathogenesis suggested an association of the GG genotype with an increased risk of SLE development and the protective role of the AA genotype [59,60]. On the other hand, a study by Park et al., utilizing a Chinese population, provided contradictory findings with regard to predisposition for RA development [61]. 

The other genetic variant investigated in the present study, *NKG2D* rs1049174, has been also previously associated with natural cytotoxic activity and susceptibility to overall cancer development [57]. The *NKG2D* rs1049174 GG homozygous genotype corresponded to higher NK activity and reduced cancer risk, while the opposite effect was ascribed to the CC genotype. These findings have been supported by a study concerning colorectal cancer [62]. Presence of the GG genotype correlated with a decreased risk of colorectal cancer development. The *NKG2D* rs1049174 polymorphism was also associated with the risk of aero-digestive tract cancer in a lifestyle-dependent manner [63]. The protective effect of the G allele in cancer development was observed among non-smoking and non-drinking individuals. Moreover, it was reported that *NKG2D* rs1049174 may affect the clinical outcome of unrelated bone marrow transplantation [64]. Patients with standard-risk diseases receiving transplants from donors possessing the GG genotype were characterized by a better overall survival after transplantation. In the present study, the *NKG2D* CC genotype was associated with a lack of response to anti-TNF therapy, while presence of the heterozygous genotype was correlated with a positive anti-TNF outcome. The functional impact of the *NKG2D* rs1049174 polymorphism on protein expression was also suggested [65]. In a study conducted by Imai et al., the GG genotype was significantly associated with increased levels of NKG2D surface expression, both in NK and CD8^+^ T cells.

In both studied *NKG2D* polymorphisms, a lack of response correlated with genetic variants that are associated with decreased NK activity. In line with these observations, the killer-cell immunoglobulin-like receptor (*KIR*) polymorphism, related to diminished cytotoxic activity of NK cells, has been linked to poor response of anti-TNF therapy in a study involving RA patients [66]. These results may imply that genetic factors contributing to an inhibitory profile of NK cells may exert a negative influence on anti-TNF treatment efficacy. On the other hand, the beneficial impact of the heterozygous genotype on clinical outcomes may suggest that balanced NKG2D activity constitutes an important issue in the context of successful anti-TNF therapy. The expression of both functional variants of the NKG2D protein, on the cells of patients with heterozygous genotype, implicates an intermediate level of activity of these receptors. It can be hypothesized that moderate signal transduction via the NKG2D pathway may result in the most adequate triggering of immune effector cells involved in mechanisms of anti-TNF response. This may act beneficially for treatment outcomes. It is equally conceivable that the advantageous influence of the heterozygous genotype on anti-TNF therapy efficacy can be attributed solely to the protein encoded by *NKG2D* rs2255336 A or rs1049174 G allele. In such a case, rs2255336 AA or rs1049174 GG homozygosity should exert the most favorable effect. Although the present study did not reveal a positive correlation of the *NKG2D* rs2255336 AA or *NKG2D* rs1049174 GG genotypes with good anti-TNF response, it is possible that this may stem from the relatively small number of patients with *NKG2D* rs2255336 AA or rs1049174 GG genotypes. In order to resolve these assumptions, further investigations with larger patient cohorts should be performed.

Data obtained in this study revealed significant associations between examined *NKG2D* genetic variants and the response to TNF inhibitors at the 12th week. With respect to the outcome of therapy, measured at the 24th week, no significant relationships were detected. However, it should be underlined that the 12th week of anti-TNF treatment constitutes an important time point of a follow-up. Assessments during the third month are crucial for further predictions of therapy outcomes. Lack of response at the 12th week is associated with a very low probability of achieving a good response by the 24th week [67,68]. On the other hand, clinical response attained at this time point acts as a good predictor for reaching remission by the 12th month [67,68]. Furthermore, many trials investigating the efficacy or safety of anti-TNF agents employ the 12th week time point as a critical decision point [69,70]. In addition, some studies documenting associations between genetic variants and clinical outcome of anti-TNF treatment at the 12th week did not reveal a significant relationship with respect to response attained after 24 weeks of therapy, or during a later follow-up [71,72,73].

The results derived from the present study may suggest that the investigated *NKG2D* genetic variants interfere with the mechanisms of action among anti-TNF agents in a time-specific manner. It is conceivable that the influence of *NKG2D* polymorphisms, in the context of response to TNF inhibitors, may be strictly related to the period of implemented treatment and might exert favorable or deleterious effect, only during the early stage of anti-TNF therapy. However, these hypotheses remain abstract and functional studies are a prerequisite to clarify the potential mechanism underlying a contribution of *NKG2D* polymorphisms to anti-TNF agents’ actions. The lack of association between anti-TNF therapy outcome at the 24th week and the examined *NKG2D* polymorphisms might be also attributable to the statistical power of the study being insufficient to detect small or moderate effects. Therefore, the results from the present study require confirmation with larger patient cohorts. It would also be important to assess the potential influence of *NKG2D* polymorphisms on the response to long-term anti-TNF treatment.

To the best of our knowledge, this is the first study investigating *NKG2D* genetic variants in the context of their potential impact on the efficacy of anti-TNF therapy. Obtained results imply a possible contribution of *NKG2D* gene variants in determining the therapeutic effect of anti-TNF agents. However, potential limitations of the present study should be considered. It cannot be ruled out that other polymorphisms in LD with the examined SNPs may contribute to the observed effects. In addition, this study was limited by a modest sample size and *p*-value cut-off < 0.05, which might be considered liberal. Results derived from this study require validation in larger patient cohorts from Caucasian populations, as well as from other ethnic groups.

In conclusion, the results derived from the present study provide the first evidence that genetic variation within the *NKG2D* gene may be involved in balancing the efficacy of anti-TNF therapy in RA patients of Caucasian origin. The presence of the heterozygous genotypes of both the *NKG2D* rs1049174 and rs2255336 in patients is positively correlated with a better response to anti-TNF treatment. On the other hand, lack of response was more frequently observed among patients homozygous for the *NKG2D* rs2255336 GG or rs1049174 CC genotypes. The present data imply that *NKG2D* polymorphisms display potential to act as pharmacogenomic biomarkers of responsiveness to anti-TNF therapy in RA.

## Figures and Tables

**Table 1:** Baseline characteristics of RA patients.

RA Patients	N = 280
Demographics	
Sex (females/males (% of females))	220/60 (78.6%)
Age (years) (mean (±SD))	51.6 (±12.3)
Current smokers (%)	33.3%
Clinical data	
Disease duration (years) (mean (±SD))	12.6 (±8.1)
Disease onset (years) (mean (±SD))	39.2 (±12.0)
DAS28 at baseline (mean (±SD))	6.5 (±0.6)
CRP at baseline (mean (±SD))	24.4 (±35.7)
RF-positive (%)	65.9%
anti-CCP+ (%)	95.4%
Anti-TNF drugs	
Etanercept	54%
Adalimumab	33%
Infliximab	7%
Certolizumab pegol	6%
Concomitant treatment	
Glucocorticosteroids	91%
Methotrexate	92%

RA: rheumatoid arthritis; DAS28: disease activity score 28; CRP: C-reactive protein; RF: rheumatoid factor; anti-CCP: anti-cyclic citrullinated peptide antibodies; SD: standard deviation; percentages calculated in regard to a total number of patients.

**Table 2:** Relationships of the *NKG2D* gene polymorphisms with EULAR responses at 12 and 24 weeks of anti-TNF therapy.

	EULAR 12 Weeks		EULAR 24 Weeks	
	No Response (Number (%))	Good/Moderate Response (Number (%))	No Response (Number (%))	Good/Moderate Response (Number (%))
***NKG2D*** **rs1049174**	(C|G: general population 51.3%|48.7%; Caucasian population: 68.4%|31.6%) *			
C	38 (82.6%) ^a^	342 (67.9%) ^a^	19 (67.9%)	348 (68.0%)
G	8 (17.4%) ^a^	162 (32.1%) ^a^	9 (32.1%)	164 (32.0%)
CC	17 (73.9%) ^b^	106 (41.2%) ^b^	6 (42.9%)	113 (44.1%)
CG	4 (17.4%) ^c^	130 (50.6%) ^c^	7 (50.0%)	122 (47.7%)
GG	2 (8.7%)	21 (8.2%)	1 (7.1%)	21 (8.2%)
***NKG2D*** **rs1154831**	(A|C: general population 10.2%|89.8%; Caucasian population: 21.2%|78.8%) *			
A	11 (23.9%)	90 (17.5%)	6 (21.4%)	90 (17.6%)
C	35 (76.1%)	424 (82.5%)	22 (78.6%)	422 (82.4%)
AA	1 (4.3%)	6 (2.3%)	1 (7.1%)	6 (2.3%)
AC	9 (39.1%)	78 (30.4%)	4 (28.6%)	78 (30.5%)
CC	13 (56.5%)	173 (67.3%)	9 (64.3%)	172 (67.2%)
***NKG2D*** **rs2255336**	(A|G: general population 23.8%|76.2%; Caucasian population: 18.6%|81.4%) *			
A	2 (4.3%) ^d^	114 (22.2%) ^d^	4 (14.3%)	109 (21.3%)
G	44 (95.7%) ^d^	400 (77.8%) ^d^	24 (85.7%)	403 (78.7%)
AA	0 (0.0%)	12 (4.7%)	0 (0.0%)	12 (4.7%)
AG	2 (8.7%) ^e^	90 (35.0%) ^e^	4 (28.6%)	85 (33.2%)
GG	21 (91.3%) ^f^	155 (60.3%) ^f^	10 (71.4%)	159 (62.1%)

^a^: *p* = 0.031, OR = 2.39, 95%CI (1.07, 6.05); ^b^: CC vs. CG + GG, *p* = 0.004, OR = 4.02, 95%CI (1.45, 12.87); ^c^: CG vs. CC + GG, *p* = 0.002, OR = 0.21, 95%CI (0.05, 0.65); ^d^: *p* = 0.002, OR = 6.26, 95%CI (1.59, 54.05); ^e^: AG vs. AA + GG, *p* = 0.010, OR = 0.18, 95%CI (0.02, 0.75); ^f^: GG vs. AG + AA, *p* = 0.003, OR = 6.88, 95%CI (1.62, 61.80); *: source: The 1000 Genomes Project (phase 3) (http://www.internationalgenome.org/); OR: odds ratio; 95%CI: 95% confidence interval; EULAR: European League Against Rheumatism response criteria; percentages calculated in regard to a total number of patients in a given responders group.

**Table 3:** Distribution of the *NKG2D* alleles and genotypes with regard to selected clinical parameters. Percentages calculated in regard to a total number of patients measured for a given feature.

	DAS28 at Baseline (Mean (±SD))	CRP at Baseline (Mean (±SD))	RF+ (Number (%))	CCP+ (Number (%))
***NKG2D*** **rs1049174**				
CC	6.58 (±0.64)	26.36 (±35.52)	80 (45.5%)	101 (43.9%)
CG	6.48 (±0.61)	21.01 (±27.02)	83 (47.2%)	108 (47.0%)
GG	6.67 (±0.58)	33.04 (±64.81)	13 (7.4%)	21 (9.1%)
***NKG2D*** **rs1154831**				
AA	6.59 (±0.53)	24.54 (±18.31)	4 (2.3%)	5 (2.2%)
AC	6.57 (±0.65)	18.14 (±18.19)	57 (32.4%)	68 (29.6%)
CC	6.52 (±0.62)	27.33 (±41.55)	115 (65.3%)	157 (68.3%)
***NKG2D*** **rs2255336**				
AA	6.85 (±0.61)	37.14 (±56.51)	6 (3.4%)	7 (3.0%)
AG	6.50 (±0.57)	24.09 (±36.97)	60 (34.1%)	77 (33.5%)
GG	6.53 (±0.65)	23.69 (±32.91)	110 (62.5%)	146 (63.5%)

**Table 4:** Relationships of the *NKG2D* gene polymorphisms with EULAR responses to adalimumab and etanercept at 12th week of the therapy

	ADA		ETA	
	No Response (Number (%))	Good/Moderate Response (Number (%))	No Response (Number (%))	Good/Moderate Response (Number (%))
***NKG2D* rs1049174**				
C	10 (71.4%)	103 (64.4%)	13 (81.3%)	153 (66.5%)
G	4 (28.6%)	57 (35.6%)	3 (18.8%)	77 (33.5%)
CC	4 (57.1%)	28 (35.0%)	6 (75%)	50 (43.5%)
CG	2 (28.6%)	47 (58.8%)	1 (12.5%)	53 (46.1%)
GG	1 (14.3%)	5 (6.3%)	1 (12.5%)	12 (10.4%)
***NKG2D* rs2255336**				
A	2 (14.3%)	34 (21.3%)	0 (0%) ^a^	57 (24.8%) ^a^
G	12 (85.7%)	126 (78.8%)	16 (100%) ^a^	173 (75.2%) ^a^
AA	0 (0%)	3 (3.8%)	0 (0%)	8 (7.0%)
AG	2 (28.6%)	28 (35.0%)	0 (0%)	41 (35.7%)
GG	5 (71.4%)	49 (61.3%)	8 (100%) ^b^	66 (57.4%) ^b^

^a^: *p*-value = 0.027, OR = 0.00, 95%CI (0.00-0.82); ^b^: GG vs. AA+AG, *p*-value = 0.021, OR = ∞, 95%CI (1.19–∞); OR: odds ratio; 95% CI: 95% confidence interval; ADA: adalimumab; ETA: etanercept EULAR: European League Against Rheumatism response criteria; percentages calculated in regard to a total number of patients in a given responders group.

**Table 5:** Relationships of the *NKG2D* haplotypes with EULAR responses at 12th and 24th week of anti-TNF therapy

	EULAR 12 Weeks		EULAR 24 Weeks	
	No Response (Number (%))	Good/Moderate Response (Number (%))	No Response (Number (%))	Good/Moderate Response (Number (%))
CGA	10 (3.6%)	80 (28.6%)	5 (1.8%)	80 (28.6%)
CGC	20 (7.1%)	225 (80.4%)	12 (4.3%)	224 (80.0%)
GAC	1 (0.4%) ^a^	92 (32.9%) ^a^	4 (1.4%)	87 (31.1%)
GGC	6 (2.1%) ^b^	143 (51.1%) ^b^	8 (2.9%)	135 (48.2%)

^a^: GAC vs. others, *p*-value = 0.001, OR = 0.08, 95%CI (0.00, 0.52); ^b^: GGC vs. others, *p*-value = 0.008, OR = 0.28, 95%CI (0.09, 0.78); haplotypes were composed from the following order: rs1049174 (C/G)–rs2255336 (A/G)–rs1154831 (C/A); OR: odds ratio; 95%CI: 95% confidence interval; EULAR: European League Against Rheumatism response criteria; percentages calculated in regard to a total number of patients.
