# Addendum: Iwaszko et al., Influence of NKG2D Genetic Variants on Response to Anti-TNF Agents in Patients with Rheumatoid Arthritis. *Genes* 2018, *9*, 64

**DOI:** 10.3390/genes9020094

**Published:** 2018-02-14

**Authors:** Milena Iwaszko, Jerzy Świerkot, Katarzyna Kolossa, Sławomir Jeka, Piotr Wiland, Katarzyna Bogunia-Kubik

**Affiliations:** 1 Laboratory of Clinical Immunogenetics and Pharmacogenetics, Hirszfeld Institute of Immunology and Experimental Therapy, Polish Academy of Sciences, Weigla 12, 53-114 Wrocław, Poland; bogunia@iitd.pan.wroc.pl; 2 Department of Rheumatology and Internal Medicine, Wrocław Medical University, Borowska 213, 50-556 Wrocław, Poland; jurekswierkot0@poczta.onet.pl (J.Ś.); pwiland1@gmail.com (P.W.); 3 Clinical Department of Rheumatology and Connective Tissue Diseases, Hospital University Number 2 Jana Biziela, Ujejskiego 75, 85-168 Bydgoszcz, Poland; katarzynakolossa@wp.pl (K.K.); s.jeka@wp.pl (S.J.); 4 Department of Internal, Occupational Diseases, Hypertension and Clinical Oncology, Wrocław Medical University, Borowska 213, 50-556 Wrocław, Poland

It has been brought to our attention that the funding of the National Center of Science (Poland) was missing in the acknowledgements section of our published paper [1], and therefore we would like to add this and report the acknowledgements as follows:
